# ClassifyCNV: a tool for clinical annotation of copy-number variants

**DOI:** 10.1038/s41598-020-76425-3

**Published:** 2020-11-23

**Authors:** Tatiana A. Gurbich, Valery Vladimirovich Ilinsky

**Affiliations:** Genotek Ltd., Nastavnicheskii pereulok 17/1, 105120 Moscow, Russia

**Keywords:** Genetics research, Clinical genetics, Genomics, Medical genetics, Genome informatics, Sequence annotation, Software

## Abstract

Copy-number variants (CNVs) are an important part of human genetic variation. They can be benign or can play a role in human disease by creating dosage imbalances and disrupting genes and regulatory elements. Accurate identification and clinical annotation of CNVs is essential, however, manual evaluation of individual CNVs by clinicians is challenging on a large scale. Here, we present ClassifyCNV, an easy-to-use tool that implements the 2019 ACMG classification guidelines to assess CNV pathogenicity. ClassifyCNV uses genomic coordinates and CNV type as input and reports a clinical classification for each variant, a classification score breakdown, and a list of genes of potential importance for variant interpretation. We validate ClassifyCNV’s performance using a set of known clinical CNVs and a set of manually evaluated variants. ClassifyCNV matches the pathogenicity category for 81% of manually evaluated variants with the significance of the remaining pathogenic and benign variants automatically determined as uncertain, requiring a further evaluation by a clinician. ClassifyCNV facilitates the implementation of the latest ACMG guidelines in high-throughput CNV analysis, is suitable for integration into NGS analysis pipelines, and can decrease time to diagnosis. The tool is available at https://github.com/Genotek/ClassifyCNV.

## Introduction

Copy-number variation is a form of structural genetic variation that involves a gain or loss of DNA segments. Copy-number variants (CNVs) are > 50 bp in size and can include a part of a gene, a whole gene, or a longer genomic region^1^. CNVs are associated with a number of genetic disorders, including autism spectrum disorders, neurodevelopmental disorders, and autoimmune diseases^2,3^. With advancements in next-generation sequencing technology and an increasing availability of bioinformatics tools to analyze NGS data, clinical labs are now able to process and detect CNVs in batches of exomes, genomes, and gene panels. In order for the patients to receive an accurate diagnosis and appropriate care, it is essential to correctly determine the pathogenicity of variants.

In late 2019 ACMG released updated guidelines for clinical classification of CNVs^4^. Each CNV is classified into one of the following categories: benign, likely benign, a variant of uncertain significance, likely pathogenic, or pathogenic. The new guidelines take into account a wide range of CNV properties and allow for comprehensive analysis and accurate classification of variants. However, implementation of the guidelines on a large scale is challenging, as each CNV requires considerable time on the part of a clinician to obtain a final pathogenicity score. Although the new guidelines are intended for manual evaluation, computational analysis expedites the process and determines the impact of CNVs more efficiently. Available CNV annotation tools use criteria that are different from the new ACMG guidelines^5–7^, hence, a new computational approach is needed.

Here, we present ClassifyCNV, a command-line tool that allows for rapid high-throughput classification of CNVs in accordance with the latest ACMG guidelines.

## Methods

### Databases

The databases used to implement the 2019 ACMG criteria for clinical classification of copy-number variants (CNVs)^4^ are listed in Supplementary Table S1. For each database we indicate which human genome build it is available for (hg19 or hg38). If a database is only available for one genome build, we used CrossMap v0.4.2^8^ and the UCSC chain files, available from the UCSC genome browser^9^, to lift over genomic coordinates between the genome builds.

All of the mentioned databases were converted to BED format and are available in the ClassifyCNV repository. We recommend that the local versions of the ClinGen databases are updated regularly by executing the update_clingen.sh script, which is available in the ClassifyCNV repository.

### Implementation

ClassifyCNV is implemented in Python 3, runs on Linux, UNIX, and Mac OS X, and requires BEDTools v.2.27.1 or higher^10^. Both the GRCh37 and the GRCh38 genome builds are supported.

**Figure 1:**
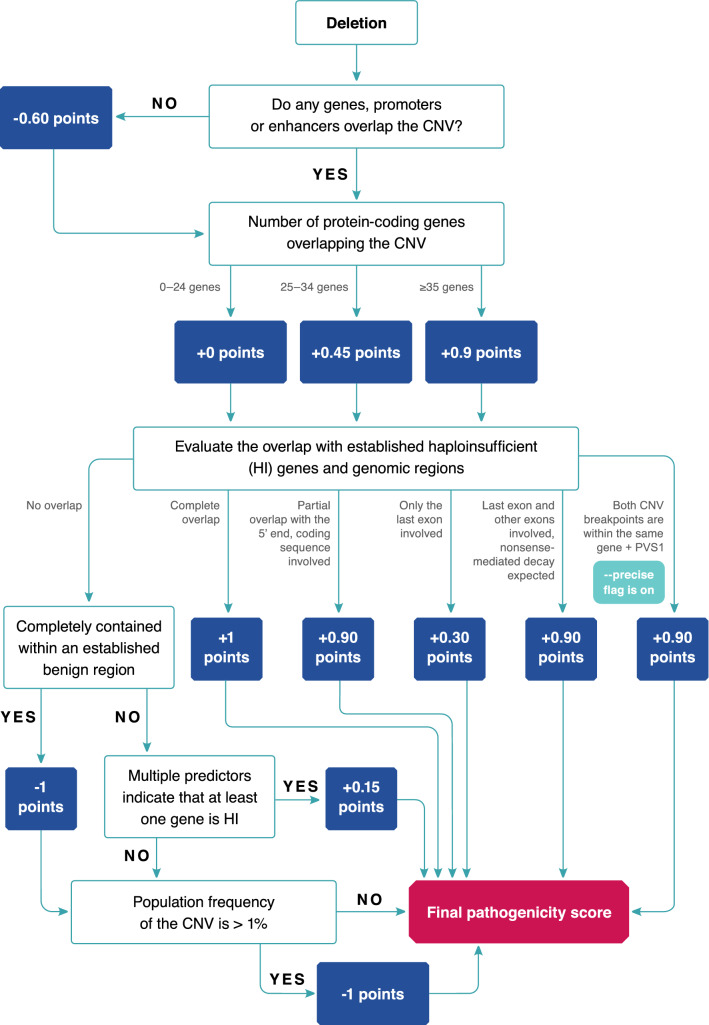
The algorithm to determine the pathogenicity score of a copy-number loss.

**Figure 2:**
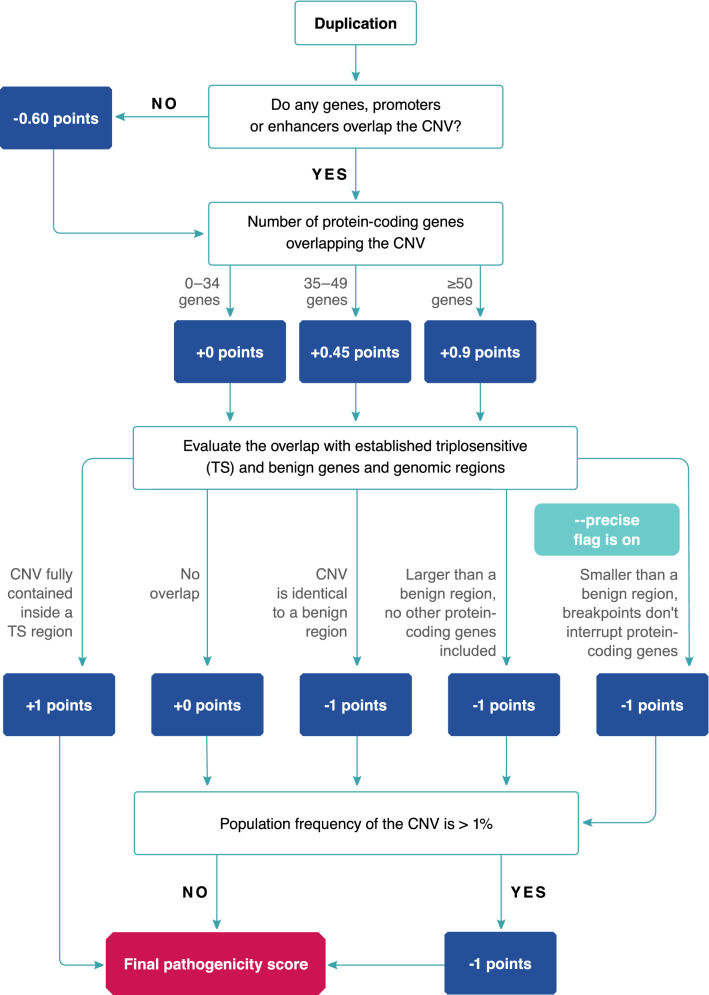
The algorithm to determine the pathogenicity score of a copy-number gain.

ClassifyCNV accepts a BED file as input and requires the user to provide genomic coordinates and type (deletion or duplication) for each CNV. ClassifyCNV does not evaluate the quality of the CNV calls as it is expected to be done during the CNV calling and filtering steps. The tool then uses the criteria described in the ACMG scoring rubrics for copy-number loss and gain^4^ to evaluate the clinical significance of the CNVs. The criteria that are implemented in ClassifyCNV are listed in Supplementary Table S2 for copy-number losses and in Supplementary Table S3 for copy-number gains. Points are awarded for each evaluated section of the rubric. Clinical classification is calculated based on the total number of points assigned to a CNV. The flowchart of the algorithm is shown in Fig. 1 for copy-number losses and in Fig. 2 for copy-number gains.

To assess the genomic content of each variant, ClassifyCNV checks for a full or partial (≥ 1 bp) overlap with protein-coding and noncoding genes, as well as enhancers and promoters. It also tracks the number of protein-coding genes that are fully or partially overlapped by each CNV. To assess whether any established dosage-sensitive genes or regions are included and what effect the deletion or duplication might have on their expression, each CNV is evaluated against a set of curated haploinsufficient and triplosensitive genes and genomic regions obtained from ClinGen^11^. A score of ‘3’ is required for a gene or genomic region to be considered haploinsufficient or triplosensitive. For partially overlapped dosage-sensitive genes ClassifyCNV evaluates which regions within the gene are involved as per ACMG guidelines. If a deletion does not encompass genes or regions that are known to be haploinsufficient, ClassifyCNV checks whether haploinsufficiency is predicted for any genes within the deletion. To satisfy this condition, a gene is required to have a DECIPHER HI index ≤ 10%^12^, a gnomAD pLI score ≥ 0.9 and the upper bound of the observed/expected confidence interval < 0.35^13^. Finally, to assess whether the CNV is likely to be benign, ClassifyCNV obtains the population frequencies of similar variants from DGV^14^ and gnomAD^15^. For each analyzed CNV that does not contain known dosage-sensitive genes or genomic regions, the population frequencies of known overlapping CNVs are extracted. An overlap of at least 80% of the query CNV length is required. If multiple known variants overlap the CNV, their average population frequency is calculated. A CNV is considered common if its population frequency is > 1%.

ClassifyCNV continues the evaluation through the end of the rubric for all CNVs, including the ones where a benign or pathogenic classification is determined before all of the conditions in the rubric have been evaluated.

ClassifyCNV outputs a tab-delimited file that can be used by another pipeline in downstream analysis or evaluated by a clinician. For each variant ClassifyCNV reports the clinical classification, the total number of points, a breakdown of how the final pathogenicity score was determined, a list of established and predicted dosage-sensitive genes encompassed by the CNV, and a list of all protein-coding genes within the CNV. As some of the sections of the ACMG scoring rubrics require manual evaluation by a clinician, the information provided can be used to continue the evaluation if necessary.

## Results

To test speed performance of ClassifyCNV, we obtained a set of 17,683 duplications and 20,805 deletions from the nstd102 study in ClinVar^16^. We used the hg19 coordinates and ran ClassifyCNV using the -precise flag, thus treating the CNV coordinates as exact. For CNVs for which precise coordinates were unknown, we used the inner coordinates. The run completed in less than 60 s on a 64-bit Linux virtual machine using two cores.

We used the same set of CNVs to evaluate the ClassifyCNV performance on clinical data. The ClinVar variants were obtained from studies published prior to 2019 and, therefore, classified before the current ACMG guidelines were released. The comparison of ClinVar and ClassifyCNV classifications is shown in Table 1.

**Table 1:** ClassifyCNV performance on ClinVar data.

ClinVar classification		ClassifyCNV classification		ClassifyCNV performance evaluation	
Classification	Count	Classification	Count (percentage)	Sensitivity	Specificity
Pathogenic/likely pathogenic	6780	Pathogenic/likely pathogenic Uncertain significance Benign/likely benign	3865 (57%) 2902 (42.8%) 13 (0.2%)	57.0%	99.0%
Benign/likely benign	19,026	Benign/likely benign Uncertain significance Pathogenic/likely pathogenic	2246 (11.8%) 16,687 (87.7%) 93 (0.5%)	11.8%	99.6%
Uncertain significance	12,682	Uncertain significance Benign/likely benign Pathogenic/likely pathogenic	12,394 (97.8%) 69 (0.5%) 219 (1.7%)	97.8%	24.1%

The pathogenic/likely pathogenic variants and variants of uncertain significance show a high degree of concordance between the original ClinVar classification and the ClassifyCNV result. The majority of benign variants were classified as variants of uncertain significance.

The pathogenic/likely pathogenic variants and variants of uncertain significance had a high degree of concordance between the original ClinVar classification and the ClassifyCNV result (57% and 97.8% respectively). The majority of benign variants were classified as variants of uncertain significance [16,687 (87.7%)]. 14,356 of these variants did not receive any points during the classification, indicating that the variants do contain genes or regulatory elements. However, the information about the genetic content within these variants was unavailable or did not strongly support reclassification of the variants from uncertain significance to benign or pathogenic. Despite the low sensitivity (11.8%) when evaluating benign variants, ClassifyCNV showed a high degree of specificity (99.6%) as the tool is conservative when moving variants between categories. Since the classification parameters used by ClassifyCNV are different from the parameters used prior to the release of the 2019 ACMG guidelines, we do not expect full concordance even when evaluating variants manually.

To assess the concordance of the ClassifyCNV calls with the results of manual evaluation we obtained the complete list of 114 variants previously classified by the ACMG/ClinGen committee using the new guidelines^4^ (Table 2, Supplementary Table S4). In the ACMG/ClinGen dataset, the manual classification results were provided by two evaluators who assessed the variants independently. We re-grouped the calls into 4 categories: pathogenic/likely pathogenic, uncertain significance, benign/likely benign, and conflicting. The latter category contained the variants that the two evaluators disagreed on. CNV breakpoints were presumed to be accurate and the -precise flag was used.

**Table 2:** Comparison of ClassifyCNV calls to the results of manual annotation by ACMG/ClinGen.

ACMG/ClinGen classification		ClassifyCNV classification		ClassifyCNV performance evaluation	
Classification	Count	Classification	Count (percentage)	Sensitivity	Specificity
Pathogenic/likely pathogenic	23	Pathogenic/likely pathogenic Uncertain significance	14 (60.9%) 9 (39.1%)	60.9%	98.4%
Benign/likely benign	8	Benign/likely benign Uncertain significance	2 (25.0%) 6 (75.0%)	25%	100%
Uncertain significance	53	Uncertain significance Pathogenic/likely pathogenic	52 (98.1%) 1 (1.9%)	98.1%	51.6%
Conflicting results	30	Uncertain significance Pathogenic/likely pathogenic	28 (93.3%) 2 (6.7%)	-	-

For 81% of CNVs, the ClassifyCNV result matched the ACMG/ClinGen result category. ClassifyCNV showed a high degree of specificity for pathogenic/likely pathogenic and benign/likely benign variants.

For 81% of CNVs, the ClassifyCNV result matched the ACMG/ClinGen category (for 76% of CNVs the match was exact and for 5% ClassifyCNV determined the CNV to be likely benign or likely pathogenic, while the manual evaluation result was benign or pathogenic, respectively). In only one case did ClassifyCNV place a variant of uncertain significance into the likely pathogenic category. The pathogenicity points were assigned due to the large number of protein-coding genes encompassed by the CNV, many of which belonged to the same gene family and thus were not counted individually during the manual evaluation. For both benign/likely benign and pathogenic/likely pathogenic categories, ClassifyCNV showed a high degree of specificity (100% and 98.4% respectively). There were no occurrences of benign/likely benign variants classified as pathogenic/likely pathogenic and vice versa. For variants automatically classified as uncertain, a manual evaluation of the published literature and patients’ family histories by a clinician was required to arrive at the final classification.

Lastly, we compared ClassifyCNV performance to the performance of AnnotSV^5^, a comprehensive annotation tool that implements an earlier version of the ACMG criteria. To compare the two tools, we used the ACMG/ClinGen manually curated set of 114 variants. We removed the variants for which the ACMG/ClinGen classification was conflicting since calculating sensitivity, specificity and accuracy for such variants would not be possible. We analyzed the remaining 84 CNVs using AnnotSV version 2.4 with default settings and ClassifyCNV with the -precise flag enabled to treat the CNV coordinates as exact. The comparison of the two tools is shown in Table 3.

**Table 3:** A comparison of ClassifyCNV and AnnotSV.

		ClassifyCNV (%)	AnnotSV (%)
Pathogenic/likely pathogenic	Sensitivity	60.9	100
	Specificity	98.4	19.7
	Accuracy	88.1	41.7
	% Benign/likely benign calls	0	0
	% Uncertain calls	39.1	0
Benign/likely benign	Sensitivity	25	37.5
	Specificity	100	92.1
	Accuracy	92.9	86.9
	% Pathogenic/likely pathogenic calls	0	62.5
	% Uncertain calls	75	0
Uncertain significance	Sensitivity	98.1	5.6
	Specificity	51.6	100
	Accuracy	81	40.5

Both tools were tested on a set of 84 variants manually evaluated by ACMG/ClinGen. While ClassifyCNV produced a higher percentage of uncertain calls compared to AnnotSV, it had higher specificity and accuracy for pathogenic/likely pathogenic and benign/likely benign variants.

Compared to ClassifyCNV, AnnotSV is less conservative when making pathogenic/likely pathogenic calls. Out of 84 variants, AnnotSV determined 72 to be pathogenic/likely pathogenic, compared to 15 calls by ClassifyCNV and 23 calls by ACMG/ClinGen manual evaluation. AnnotSV showed higher sensitivity for pathogenic/likely pathogenic variants (100% vs 60.9% by ClassifyCNV) and benign/likely benign variants (37.5% vs 25% by ClassifyCNV). However, both the specificity and the accuracy of AnnotSV were lower. For benign/likely benign variants ClassifyCNV had 100% specificity and 92.9% accuracy, while AnnotSV’s values were 92.1% and 86.9%, respectively. For pathogenic/likely pathogenic variants ClassifyCNV had 98.4% specificity while AnnotSV’s specificity was 19.7%. The accuracy of ClassifyCNV and AnnotSV was 88.1% and 41.7%, respectively.

In summary, while ClassifyCNV places variants in the uncertain category more often compared to AnnotSV, the high specificity and accuracy of ClassifyCNV make it a more suitable tool for evaluation of CNVs using the latest ACMG/ClinGen guidelines. A follow-up evaluation by a clinician is expected to refine the classification of variants of uncertain significance.

## Discussion

ClassifyCNV is the first tool that automates the implementation of the updated ACMG guidelines to classify CNVs. It produces a rapid and reliable evaluation of variants and is suitable for high-throughput analysis. The tool can be easily integrated into existing pipelines and can expedite the evaluation of CNVs, helping to reduce the time to diagnosis.

ClassifyCNV errs on the side of caution when moving a variant between categories, as advised by the new ACMG guidelines. Therefore, if convincing data are not available, a CNV is likely to remain a variant of uncertain significance. Although a follow-up evaluation by a clinician may be necessary for these variants, ClassifyCNV significantly facilitates the process by completing the evaluation of gene content, dosage-sensitivity, and population frequencies and outputting a list of genes of interest.

## Supplementary information

Supplementary Information.

## Data Availability

All external datasets are described and cited in Supplementary Table S1 and in the manuscript. All data used by ClassifyCNV are available at https://github.com/Genotek/ClassifyCNV.
