# Supplementary material for: ClassifyCNV: a tool for clinical annotation of copy-number variants
Source: Sci Rep. 2020 Nov 23;10:20375. doi: 10.1038/s41598-020-76425-3 (PMC7683568; doi:10.1038/s41598-020-76425-3)
Supplement: Supplementary file 1 — Supplementary Information. [file 41598_2020_76425_MOESM1_ESM.pdf]

ClassifyCNV: a tool for clinical annotation of copy-number variants

Tatiana A. Gurbich and Valery Vladimirovich Ilinsky

Supplementary Information

Supplementary Table S1. Databases used by ClassifyCNV.

**Database**	**Genome build**	**Sections of the Copy Number Loss rubric where the database is used**	**Sections of the Copy Number Gain rubric where the database is used**	**Links and citations**	**Comment**
RefGene	hg19, hg38	1, 3, 2E	1, 3, 2D, 2E, 2F, 2G	http://hgdownload.cse.ucsc.edu/goldenPath/hg19/database/refGene.txt.gz<br><br>http://hgdownload.cse.ucsc.edu/goldenPath/hg38/database/refGene.txt.gz<br><br>(O'Leary et al., 2016)[1]	Only one transcript per gene was kept. A MANE Select transcript was used if available. Otherwise, the longest transcript was used.
Promoters	hg19, hg38	1	1	-	The promoter database was generated by obtaining the coordinates of the 500bp region directly upstream of every gene in the refGene database.
VISTA enhancers	hg19	1	1	https://enhancer.lbl.gov/cgi-bin/imagedb3.pl?page_size=10((0;show=1;search.result=yes;page=1;form=search;search.form=no;action=search;search.sequence=1<br><br>(Visel et al., 2007)[2]	The database was pre-parsed to only keep human enhancers.<br><br>This database is combined with FANTOM5 enhancers and enhancers from the Ensembl regulatory build to make the Enhancers.3sources.merged.bed file.
FANTOM5 enhancers	hg19, hg38	1	1	https://fantom.gsc.riken.jp/5/datafiles/latest/extra/Enhancers/human_permissive_enhancers_phase_1_and_2.bed.gz<br><br>https://fantom.gsc.riken.jp/5/datafiles/reprocessed/hg38_latest/extra/enhancer/F5.hg38.enhancers.bed.gz<br><br>(Andersson et al., 2014)[3]	This database is combined with VISTA enhancers and enhancers from the Ensembl regulatory build to make the Enhancers.3sources.merged.bed file.
Ensembl regulatory build	hg38	1	1	ftp://ftp.ensembl.org/pub/release-100/regulation/homo_sapiens/homo_sapiens.GRCh38.Regulatory_Build.regulatory_features.20190329.gff.gz<br><br>(Zerbino et al., 2015)[4]	Only enhancers were extracted from the database.<br><br>This database is combined with VISTA enhancers and FANTOM5 enhancers to make the Enhancers.3sources.merged.bed file.

refGene gene features (5'UTR, 3'UTR, exons, cds)	hg19, hg38	2C, 2D, 2E	2E	http://genome.ucsc.edu/cgi-bin/hgTables<br><br>(Kuhn et al., 2013)[5]	Gene feature coordinates were obtained for protein-coding genes. Only one transcript per gene was used (MANE Select if available, the longest one otherwise).
ClinGen haploinsufficient and triplosensitive genes and curated regions	hg19, hg38	2A-2G	2A-2H	ftp://ftp.clinicalgenome.org/<br><br>(Rehm et al., 2015)[6]	The script that downloads and parses the databases (update_clingen.sh) is included in the repository.
DECIPHER HI Predictions Version3	hg38	2H		https://decipher.sanger.ac.uk/files/downloads/HI_Predictions_Version3.bed.gz<br><br>(Firth et al., 2009)[7]	
ExAC pLI scores	hg19	2H		ftp://ftp.broadinstitute.org/pub/ExAC_release/release1/manuscript_data/forweb_cleaned_exac_r03_march16_z_data_pLI.txt.gz<br><br>(Lek et al., 2016)[8]	
the pLoF observed/expected upper fraction (LOEUF)	hg19	2H		gs://gnomad-public/release/2.1.1/constraint/gnomad.v2.1.1.lof_metrics.by_gene.txt.bgz<br><br>(Collins et al., 2020)[9]	
DGV Gold Standard Variants (2016-05-15)	hg19, hg38	4O	4O	http://dgv.tcag.ca/dgv/docs/DGV.GS.March2016.50percent.GainLossSep.Final.hg19.gff3<br><br>http://dgv.tcag.ca/dgv/docs/DGV.GS.hg38.gff3<br><br>(MacDonald et al., 2014)[10]	Included in population_freqs.bed
gnomAD structural variant frequencies	hg19	4O	4O	https://storage.googleapis.com/gnomad-public/papers/2019-sv/gnomad_v2.1_sv.sites.bed.gz<br><br>(Collins et al., 2020)[9]	Included in population_freqs.bed

Supplementary Table S2. Implementation of the ACMG/ClinGen Copy Number Loss rubric.

Section 1: Initial assessment of genomic content

Evidence type	Evidence	The number of points suggested by the ACMG/Clingen guidelines	The number of points ClassifyCNV assigns if the condition is satisfied	Implementation
Copy-number loss content	**1A.** Contains protein-coding or other known functionally important elements.	0	0	Implemented
	**1B.** Does NOT contain protein-coding or any known functionally important elements.	−0.60	−0.60	Implemented

Section 2: Overlap with established/predicted haploinsufficiency (HI) or established benign genes/genomic regions ***(Skip to section 3 if your copy-number loss DOES NOT overlap these types of genes/regions)***

Overlap with ESTABLISHED HI genes or genomic regions and consideration of reason for referral	**2A.** Complete overlap of an established HI gene/genomic region.	1	1	Implemented
	**2B.** Partial overlap of an established HI genomic region<br>• The observed CNV does NOT contain the known causative gene or critical region for this established HI genomic region OR<br>• Unclear if known causative gene or critical region is affected OR<br>• No specific causative gene or critical region has been established for this HI genomic region	0	0	Implemented
	**2C.** Partial overlap with the 5' end of an established HI gene (3' end of the gene not involved)…		See categories below	
	**2C-1.** …and coding sequence is involved	0.90 (Range: 0.45 to 1.00)	0.90	Implemented
	**2C-2.** …and only the 5' UTR is involved	0 (Range: 0 to 0.45)	0	Implemented

	**2D.** Partial overlap with the 3’ end of an established HI gene (5’ end of the gene not involved)…		See categories below	
	**2D-1.** …and only the 3’ untranslated region is involved.	0	0	Implemented
	**2D-2.** …and only the last exon is involved. Other established pathogenic variants have been reported in this exon.	0.90 (Range: 0.45 to 0.90)	0.30	Partially implemented.<br><br>0.30 points are assigned in all cases where only the last exon is involved regardless of whether there are established pathogenic variants in this exon.
	**2D-3.** …and only the last exon is involved. No other established pathogenic variants have been reported in this exon.	0.30 (Range: 0 to 0.45)	0.30	
	**2D-4**. …and it includes other exons in addition to the last exon. Nonsense-mediated decay is expected to occur.	0.90 (Range: 0.45 to 1.00)	0.90	Implemented
	**2E.** Both breakpoints are within the same gene (intragenic CNV; gene-level sequence variant).	See ClinGen SVI working group PVS1 specifications<br>• PVS1 = 0.90<br>*(Range: 0.45 to 0.90)*<br>• PVS1_Strong = 0.45<br>*(Range: 0.30 to 0.90)*<br>• PVS1_Moderate *or* PM4 (in-frame indels) = 0.30<br>*(Range: 0.15 to 0.45)*<br>• PVS1_Supporting = 0.15<br>*(Range: 0 to 0.30)*<br>• N/A = No points, but continue evaluation	• PVS1 = 0.90<br>• N/A = No points, but continue evaluation	Partially implemented.<br><br>If the --precise flag is on, points are assigned for intragenic variants in biologically-relevant transcripts that disrupt the reading frame and are predicted to lead to nonsense-mediated decay. No points are assigned for other types of intragenic deletions.
	**2F.** Completely contained within an established benign CNV region.	−1	−1	Implemented
	**2G**. Overlaps an established benign CNV, but includes additional genomic material.	0	0	Implemented

	**2H.** Two or more HI predictors suggest that AT LEAST ONE gene in the interval is HI.	0.15	0.15	Implemented

Section 3: Evaluation of gene number

Number of protein-coding RefSeq genes wholly or partially included in the copy-number loss	**3A.** 0–24 genes	0	0	Implemented (Note: if genes belong to the same gene family, each gene within the family is counted)
	**3B.** 25–34 genes	0.45	0.45	Implemented (Note: if genes belong to the same gene family, each gene within the family is counted)
	**3C.** 35+ genes	0.90	0.90	Implemented (Note: if genes belong to the same gene family, each gene within the family is counted)

Section 4: Detailed evaluation of genomic content using cases from published literature, public databases, and/or internal lab data*(Skip to section 5 if either your CNV overlapped with an established HI gene/region in section 2, OR there have been no reports associating either the CNV or any genes within the CNV with human phenotypes caused by loss of function [LOF] or copy-number loss)*

Individual case evidence — de novo occurrences	Reported proband (from literature, public databases, or internal lab data) has either:<br>• A complete deletion of or a LOF variant within gene encompassed by the observed copy-number loss OR<br>• An overlapping copy-number loss similar in genomic content to the observed copy-number loss AND…	See categories below		
	**4A**. …the reported phenotype is highly specific and relatively unique to the gene or genomic region,	Confirmed de novo: 0.45 points each Assumed de novo: 0.30 points each (range: 0.15 to 0.45). 0.90 (total)	-	Not implemented
	**4B.** …the reported phenotype is consistent with the gene/genomic region, is highly specific, but not necessarily unique	Confirmed de novo: 0.30 points each Assumed de novo: 0.15 point each (range: 0 to 0.45)	-	Not implemented

	to the gene/genomic region.			
	**4C.** …the reported phenotype is consistent with the gene/genomic region, but not highly specific and/or with high genetic heterogeneity.	Confirmed de novo: 0.15 point each Assumed de novo: 0.10 point each (range: 0 to 0.30)	-	Not implemented
Individual case evidence — inconsistent phenotype	**4D.** …the reported phenotype is NOT consistent with what is expected for the gene/genomic region or not consistent in general.	0 points each (range: 0 to −0.30). −0.30 (total)	-	Not implemented
Individual case evidence — unknown inheritance	**4E.** Reported proband has a highly specific phenotype consistent with the gene/genomic region, but the inheritance of the variant is unknown.	0.10 points each (range: 0 to 0.15). 0.30 (total)	-	Not implemented
Individual case evidence — segregation among similarly affected family members	**4F.** 3–4 observed segregations	0.15 (0.45 max)	-	Not implemented
	**4G.** 5–6 observed segregations	0.30	-	Not implemented
	**4H.** 7 or more observed segregations	0.45	-	Not implemented
Individual case evidence — nonsegregations	**4I.** Variant is NOT found in another individual in the proband’s family AFFECTED with a consistent, specific, well-defined phenotype (no known phenocopies).	−0.45 points per family (range: 0 to −0.45). −0.90 (total)	-	Not implemented
	**4J.** Variant IS found in another individual in the proband’s family UNAFFECTED with the specific, well-defined phenotype observed in the proband.	−0.30 points per family (range: 0 to −0.30). −0.90 (total)	-	Not implemented

	**4K.** Variant IS found in another individual in the proband's family UNAFFECTED with the nonspecific phenotype observed in the proband.	−0.15 points per family (range: 0 to −0.15). −0.30 (total)	-	Not implemented
Case–control and population evidence	**4L.** Statistically significant increase amongst observations in cases (with a consistent, specific, well-defined phenotype) compared with controls.	0.45 per study (range: 0 to 0.45 per study). 0.45 (total)	-	Not implemented
	**4M.** Statistically significant increase amongst observations in cases (without a consistent, nonspecific phenotype OR unknown phenotype) compared with controls.	0.30 per study (range: 0 to 0.30 per study). 0.45 (total)	-	Not implemented
	**4N.** No statistically significant difference between observations in cases and controls.	−0.90 (per study) (range: 0 to −0.90 per study). −0.90 (total)	-	Not implemented
	**4O.** Overlap with common population variation.	−1 (range: 0 to −1)	−1	Implemented

Section 5: Evaluation of inheritance pattern/family history for patient being studied

Observed copy-number loss is de novo	**5A.** *Use appropriate category from de novo scoring section in section 4.*	*Use de novo scoring categories from section 4 (4A–4D) to determine score (0.45 max)*	-	Not implemented
Observed copy-number loss is inherited	**5B.** Patient with specific, well-defined phenotype and no family history. CNV is inherited from an apparently unaffected parent.	−0.30 (range: 0 to −0.45)	-	Not implemented
	**5C.** Patient with nonspecific phenotype and no family history. CNV is inherited from an apparently unaffected parent.	−0.15 (range: 0 to −0.30)	-	Not implemented

	**5D.** CNV segregates with a consistent phenotype observed in the patient's family.	*Use segregation scoring categories from section 4 (4F–4H) to determine score (0.45 max)*	-	Not implemented
Observed copy-number loss — nonsegregations	**5E.** *Use appropriate category from nonsegregation section in section 4.*	*Use nonsegregation scoring categories from section 4 (4I–4K) to determine score (–0.45 max)*	-	Not implemented
Other	**5F.** Inheritance information is unavailable or uninformative.	0	-	Not implemented
	**5G.** Inheritance information is unavailable or uninformative. The patient phenotype is nonspecific, but is consistent with what has been described in similar cases.	0.10 (range: 0 to 0.15)	-	Not implemented
	**5H.** Inheritance information is unavailable or uninformative. The patient phenotype is highly specific and consistent with what has been described in similar cases.	0.30 (range: 0 to 0.30)	-	Not implemented

Supplementary Table S3. Implementation of the ACMG/ClinGen Copy Number Gain rubric.

Section 1: Initial assessment of genomic content

Evidence type	Evidence	The number of points suggested by the ACMG/Clingen guidelines	The number of points ClassifyCNV assigns if the condition is satisfied	Implementation
Copy-number gain content	**1A.** Contains protein-coding or other known functionally important elements.	0	0	Implemented
	**1B.** Does NOT contain protein-coding or any known functionally important elements.	−0.60	−0.60	Implemented

Section 2: Overlap with established triplosensitive (TS), haploinsufficient (HI), or benign genes or genomic regions ***(Skip to section 3 if the copy-number gain DOES NOT overlap these types of genes/regions)***

Overlap with ESTABLISHED TS genes or genomic regions	**2A.** Complete overlap; the TS gene or minimal critical region is fully contained within the observed copy-number gain.	1	1	Implemented
	**2B.** Partial overlap of an established TS region<br>• The observed CNV does NOT contain the known causative gene or critical region for this established TS genomic region OR<br>• Unclear if known causative gene or critical region is affected OR<br>• No specific causative gene or critical region has been established for this TS genomic region	0	0	Implemented
Overlap with ESTABLISHED benign copy-number gain genes or genomic regions	**2C.** Identical in gene content to the established benign copy-number gain.	−1	−1	Implemented
	**2D.** Smaller than established benign copy-number gain, breakpoint(s) does not interrupt protein-	−1	−1	Implemented (only when the --precise flag is on)

	coding genes.			
	**2E.** Smaller than established benign copy-number gain, breakpoint(s) potentially interrupts protein-coding gene.	0	0	Implemented
	**2F.** Larger than known benign copy-number gain, does not include additional protein-coding genes.	−1 (range: 0 to −1.00)	−1	Implemented
	**2G.** Overlaps a benign copy-number gain but includes additional genomic material.	0	0	Implemented
Overlap with ESTABLISHED HI gene(s)	**2H.** HI gene fully contained within observed copy-number gain.	0	0	Implemented
Breakpoint(s) within ESTABLISHED HI genes	**2I.** Both breakpoints are within the same gene (gene-level sequence variant, possibly resulting in loss of function [LOF]).	See ClinGen SVI working group PVS1 specifications<br>• PVS1 = 0.90 (Range: 0.45 to 0.90)<br>• PVS1_Strong = 0.45 (Range: 0.30 to 0.90)<br>• N/A = 0 (Continue evaluation)		Not implemented
	**2J.** One breakpoint is within an established HI gene, patient's phenotype is either inconsistent with what is expected for LOF of that gene OR unknown.	0	0	Implemented
	**2K.** One breakpoint is within an established HI gene, patient's phenotype is highly specific and consistent with what is expected for LOF of that gene.	0.45	-	Not implemented
Breakpoints within other gene(s)	**2L.** One or both breakpoints are within gene(s) of no established clinical significance.	0	0	Implemented

Section 3: Evaluation of gene number

Number of protein-coding RefSeq genes wholly or partially included in the copy-number gain	**3A.** 0–34 genes	0	0	Implemented (Note: if genes belong to the same gene family, each gene within the family is counted)
	**3B.** 35–49 genes	0.45	0.45	Implemented (Note: if genes belong to the same gene family, each gene within the family is counted)
	**3C.** 50+ genes	0.90	0.90	Implemented (Note: if genes belong to the same gene family, each gene within the family is counted)

Section 4: Detailed evaluation of genomic content using cases from published literature, public databases, and/or internal lab data*(Note: If there have been no reports associating either the copy-number gain or any of the genes therein with human phenotypes caused by triplosensitivity, skip to section 5)*

Individual case evidence — de novo occurrences	Reported proband (from literature, public databases, or internal lab data) has either:<br>• complete duplication of one or more genes within the observed copy-number gain OR<br>• an overlapping copy-number gain similar in genomic content to the observed copy-number gain AND…	See categories below		
	**4A.** …the reported phenotype is highly specific and relatively unique to the gene or genomic region.	Confirmed de novo: 0.45 points each Assumed de novo: 0.30 points each (range: 0.15 to 0.45). 0.90 (total)	-	Not implemented
	**4B.** …the reported phenotype is consistent with the gene/genomic region, is highly specific, but not necessarily unique to the gene/genomic region.	Confirmed de novo: 0.30 points each Assumed de novo: 0.15 point each (range: 0 to 0.45)	-	Not implemented
	**4C.** …the reported phenotype is consistent with the gene/genomic	Confirmed de novo: 0.15 point each Assumed de novo: 0.10 point each	-	Not implemented

	region, but not highly specific and/or with high genetic heterogeneity.	(range: 0 to 0.30)		
Individual case evidence — inconsistent phenotype	**4D.** …the reported phenotype is NOT consistent with the gene/genomic region or not consistent in general.	0 points each (range: 0 to −0.30). −0.30 (total)	-	Not implemented
Individual case evidence — unknown inheritance	**4E.** Reported proband has a highly specific phenotype consistent with the gene/genomic region, but the inheritance of the variant is unknown.	0.10 points each (range: 0 to 0.15). 0.30 (total)	-	Not implemented
Individual case evidence — segregation among similarly affected family members	**4F.** 3–4 observed segregations	0.15 (0.45 max)	-	Not implemented
	**4G.** 5–6 observed segregations	0.30	-	Not implemented
	**4H.** 7 or more observed segregations	0.45	-	Not implemented
Individual case evidence — nonsegregations	**4I.** Variant is NOT found in another individual in the proband's family AFFECTED with a consistent, specific, well-defined phenotype (no known phenocopies).	−0.45 points per family (range: 0 to −0.45). −0.90 (total)	-	Not implemented
	**4J.** Variant IS found in another individual in the proband's family UNAFFECTED with the specific, well-defined phenotype observed in the proband.	−0.30 points per family (range: 0 to −0.30). −0.90 (total)	-	Not implemented
	**4K.** Variant IS found in another individual in the proband's family UNAFFECTED with the nonspecific phenotype observed in the proband.	−0.15 points per family (range: 0 to −0.15). −0.30 (total)	-	Not implemented

Case–control and population evidence	**4L.** Statistically significant increase amongst observations in cases (with a consistent, specific, well-defined phenotype) compared with controls.	0.45 per study (range: 0 to 0.45 per study). 0.45 (total)	-	Not implemented
	**4M.** Statistically significant increase amongst observations in cases (without a consistent, nonspecific phenotype OR unknown phenotype) compared with controls.	0.30 per study (range: 0 to 0.30 per study). 0.45 (total)	-	Not implemented
	**4N.** No statistically significant difference between observations in cases and controls.	−0.90 (per study) (range: 0 to −0.90 per study). −0.90 (total)	-	Not implemented
	**4O.** Overlap with common population variation.	−1 (range: 0 to −1)	−1	Implemented

Section 5: Evaluation of inheritance pattern/family history for patient being studied

Observed copy-number gain is de novo	**5A.** *Use appropriate category from de novo scoring section in section 4.*	*Use de novo scoring categories from section 4 (4A–4D) to determine score (0.45 max)*	-	Not implemented
Observed copy-number loss is inherited	**5B.** Patient with specific, well-defined phenotype and no family history. Copy-number gain is inherited from an apparently unaffected parent.	−0.30 (range: 0 to −0.45)	-	Not implemented
	**5C.** Patient with nonspecific phenotype and no family history. Copy-number gain is inherited from an apparently unaffected parent.	−0.15 (range: 0 to −0.30)	-	Not implemented
	**5D.** CNV segregates with consistent phenotype observed in the patient's family.	*Use segregation scoring categories from section 4 (4F–4H) to determine score (0.45 max)*	-	Not implemented

Observed copy-number gain — nonsegregations	**5E.** *Use appropriate category from nonsegregation section in section 4.*	*Use nonsegregation scoring categories from section 4 (4I–4K) to determine score (–0.45 max)*	-	Not implemented
	**5F.** Inheritance information is unavailable or uninformative.	0	-	Not implemented
	**5G.** Inheritance information is unavailable or uninformative. The patient phenotype is nonspecific, but is consistent with what has been described in similar cases.	0.10 (range: 0 to 0.15)	-	Not implemented
	**5H.** Inheritance information is unavailable or uninformative. The patient phenotype is highly specific and consistent with what has been described in similar cases.	0.15 (range: 0 to 0.30)	-	Not implemented

Supplementary Table S4. Comparison of ClassifyCNV calls to the results of manual annotation by ACMG/Clingen. All coordinates are hg19. For 76% of the variants, ClassifyCNV matched the ACMG/Clingen category exactly. For an additional 5% of the variants, ClassifyCNV called a variant likely benign or likely pathogenic, while the ACMG/ClinGen evaluation determined the variant to be benign or pathogenic, respectively.

Chromosome	Start position	End position	CNV type	ACMG/ClinGen Evaluator 1	ACMG/ClinGen Evaluator 2	Conflict in ACMG/ Clingen evaluation	ClassifyCNV classification	Classify CNV score
1	247,815,979	248,609,997	DEL	Likely Benign	Likely Benign		Uncertain	0.45
3	12,630,912	12,818,103	DUP	Benign	Likely Benign		Uncertain	0
7	33,347,521	33,580,567	DEL	Benign	Likely Benign		Uncertain	0
13	23,533,358	24,958,572	DUP	Benign	Likely Benign		Uncertain	0
16	6,819,602	7,026,403	DEL	Benign	Benign		Uncertain	0.15
20	14,928,598	15,134,187	DEL	Benign	Likely Benign		Uncertain	0
X	2,766,830	2,800,818	DEL	Benign	Benign		Benign	-1
X	6,455,151	8,135,644	DUP	Likely Benign	Benign		Benign	-1
1	146,500,972	147,828,089	DEL	Pathogenic	Pathogenic		Pathogenic	1
2	51,002,928	51,512,609	DEL	Not Completed	Pathogenic		Likely pathogenic	0.9
2	200,197,135	200,242,625	DEL	Pathogenic	Pathogenic		Uncertain	0
2	215,772,556	220,450,837	DUP	Likely Pathogenic	Likely Pathogenic		Likely pathogenic	0.9
2	231,309,745	242,783,384	DEL	Pathogenic	Pathogenic		Pathogenic	>1
10	4,646,926	47,531,169	DUP	Pathogenic	Pathogenic		Likely pathogenic	0.9
10	26,524,127	30,285,671	DEL	Pathogenic	Pathogenic		Pathogenic	1
10	89,653,447	89,693,702	DEL	Pathogenic	Pathogenic		Likely pathogenic	0.9
12	116,533,732	116,632,392	DUP	Likely Pathogenic	Likely Pathogenic		Uncertain	0
13	39,428,367	43,608,103	DEL	Pathogenic	Likely Pathogenic		Uncertain	0.15
13	101,956,482	102,666,953	DEL	Likely Pathogenic	Likely Pathogenic		Uncertain	0
14	101,354,421	101,489,161	DEL	Benign	Likely Pathogenic	yes	Uncertain	0
15	31,073,735	32,446,830	DEL	Pathogenic	Pathogenic		Pathogenic	1
15	37,287,175	37,788,218	DEL	Pathogenic	Pathogenic		Uncertain	0.15
15	74,398,119	76,053,391	DUP	Pathogenic	Pathogenic		Uncertain	0.45
16	3,878,449	4,046,709	DEL	Pathogenic	Pathogenic		Likely pathogenic	0.9
16	15,509,406	16,312,952	DUP	Likely Benign	Pathogenic	yes	Uncertain	0
16	15,492,307	16,041,598	DEL	Pathogenic	Likely Pathogenic		Uncertain	0.15
16	29,517,698	30,177,240	DUP	Pathogenic	Likely Pathogenic		Uncertain	0
16	29,567,295	30,226,930	DEL	Pathogenic	Pathogenic		Pathogenic	>1
16	50,031,502	51,518,183	DEL	Pathogenic	Pathogenic		Pathogenic	>1
18	53,290,008	53,383,013	DEL	Pathogenic	Likely Pathogenic		Uncertain	0
22	18,661,724	21,561,514	DUP	Pathogenic	Pathogenic		Pathogenic	>1

X	6,449,752	8,135,644	DEL	Pathogenic	Pathogenic		Pathogenic	1
X	102,857,802	103,135,921	DUP	Pathogenic	Pathogenic		Pathogenic	1
1	11,938,131	15,167,547	DEL	Uncertain	Uncertain		Likely pathogenic	0.9
1	158,461,562	159,002,778	DEL	Uncertain	Not Completed		Uncertain	0
1	174,191,836	174,957,575	DEL	Uncertain	Uncertain		Uncertain	0
2	12,770	1,522,939	DEL	Uncertain	Uncertain		Uncertain	0
2	2,143,654	2,204,279	DEL	Benign	Uncertain	yes	Uncertain	0
2	9,558,552	10,930,819	DEL	Uncertain	Uncertain		Uncertain	0
2	47,433,147	47,827,310	DUP	Uncertain	Uncertain		Uncertain	0
2	57,641,400	60,339,585	DEL	Uncertain	Uncertain		Uncertain	0
2	106,878,800	108,518,292	DUP	Likely Benign	Uncertain	yes	Uncertain	0
2	111,388,618	113,127,751	DEL	Pathogenic	Uncertain	yes	Uncertain	0
2	149,218,583	149,933,863	DUP	Uncertain	Likely Pathogenic	yes	Uncertain	0
3	10,833,963	11,406,520	DEL	Uncertain	Likely Pathogenic	yes	Pathogenic	1
3	13,019,290	13,847,073	DUP	Uncertain	Uncertain		Uncertain	0
3	24,331,917	24,453,796	DEL	Uncertain	Uncertain		Uncertain	0.15
3	76,434,692	79,133,755	DEL	Uncertain	Uncertain		Uncertain	0.15
3	123,042,165	123,477,576	DUP	Uncertain	Uncertain		Uncertain	0
3	136,422,030	137,892,515	DEL	Uncertain	Not Completed		Uncertain	0.15
4	190,131,512	190,957,473	DEL	Likely Benign	Uncertain	yes	Uncertain	0
5	36,961,572	37,789,193	DUP	Uncertain	Pathogenic	yes	Uncertain	0
5	107,249,962	108,344,036	DUP	Uncertain	Uncertain		Uncertain	0
5	127,739,699	127,871,574	DEL	Likely Pathogenic	Uncertain	yes	Uncertain	0
5	178,291,553	179,343,716	DUP	Uncertain	Uncertain		Uncertain	0
6	16,524,862	16,671,871	DEL	Uncertain	Uncertain		Uncertain	0
6	80,573,500	83,983,729	DUP	Likely Pathogenic	Uncertain	yes	Uncertain	0
6	126,419,160	128,801,386	DEL	Uncertain	Uncertain		Uncertain	0.15
6	170,575,101	170,881,749	DUP	Uncertain	Uncertain		Uncertain	0
7	70,039,743	70,188,336	DUP	Pathogenic	Uncertain	yes	Uncertain	0
7	73,774,057	74,613,903	DEL	Uncertain	Likely Pathogenic	yes	Uncertain	0
7	83,782,434	84,876,096	DUP	Uncertain	Uncertain		Uncertain	0
7	153,700,272	155,224,288	DEL	Uncertain	Pathogenic	yes	Uncertain	0
8	3,571,948	4,900,891	DUP	Uncertain	Benign	yes	Uncertain	0
8	62,538,732	65,591,265	DUP	Uncertain	Uncertain		Uncertain	0
9	203,861	378,086	DEL	Benign	Uncertain	yes	Uncertain	0
9	4,947,509	5,712,780	DEL	Uncertain	Uncertain		Uncertain	0.15
9	6,704,474	10,486,872	DUP	Uncertain	Uncertain		Uncertain	0
9	36,360,269	36,950,301	DUP	Uncertain	Uncertain		Uncertain	0
9	104,940,824	105,845,122	DEL	Uncertain	Uncertain		Uncertain	0
9	118,602,947	121,246,861	DEL	Uncertain	Uncertain		Uncertain	0.15
9	140,573,688	140,685,407	DUP	Uncertain	Pathogenic	yes	Uncertain	0
10	81,603,169	81,976,925	DEL	Uncertain	Uncertain		Uncertain	0
11	31,409,742	31,679,570	DEL	Uncertain	Uncertain		Uncertain	0
11	55,030,214	55,386,217	DEL	Benign	Uncertain	yes	Uncertain	0

11	102,948,591	103,694,905	DUP	Uncertain	Uncertain		Uncertain	0
11	126,297,655	127,275,723	DUP	Uncertain	Uncertain		Uncertain	0
12	1,740,520	3,600,867	DUP	Uncertain	Uncertain		Uncertain	0
12	62,290,628	63,836,571	DEL	Uncertain	Uncertain		Uncertain	0.15
12	112,844,056	113,841,611	DUP	Likely Pathogenic	Uncertain	yes	Uncertain	0
13	20,808,574	21,475,963	DEL	Uncertain	Uncertain		Uncertain	0
13	22,952,407	25,406,588	DUP	Uncertain	Uncertain		Uncertain	0
13	32,943,827	32,945,580	DUP	Uncertain	Uncertain		Uncertain	0
14	24,080,270	24,631,608	DUP	Uncertain	Uncertain		Uncertain	0
14	77,935,939	78,636,492	DUP	Uncertain	Uncertain		Uncertain	0
14	84,325,247	89,218,538	DEL	Uncertain	Likely Benign	yes	Uncertain	0
14	93,710,666	93,935,402	DEL	Uncertain	Uncertain		Uncertain	0
15	24,487,787	25,171,720	DUP	Pathogenic	Uncertain	yes	Uncertain	0
15	28,958,779	30,370,018	DEL	Uncertain	Pathogenic	yes	Uncertain	0.15
15	63,244,929	63,841,049	DEL	Uncertain	Uncertain		Uncertain	0
15	79,694,215	80,368,818	DUP	Uncertain	Uncertain		Uncertain	0
15	93,510,242	96,199,396	DEL	Uncertain	Likely Pathogenic	yes	Likely pathogenic	0.9
15	100,692,803	102,260,994	DUP	Uncertain	Uncertain		Uncertain	0
16	14,594,908	14,766,618	DUP	Uncertain	Pathogenic	yes	Uncertain	0
16	28,819,028	29,051,191	DUP	Uncertain	Uncertain		Uncertain	0
16	29,802,654	30,177,240	DUP	Uncertain	Likely Pathogenic	yes	Uncertain	0
16	46,503,192	49,658,927	DEL	Uncertain	Uncertain		Uncertain	0.15
16	89,727,432	89,886,148	DUP	Uncertain	Uncertain		Uncertain	0
17	7,448,219	7,573,899	DUP	Uncertain	Uncertain		Uncertain	0
17	29,681,300	29,768,428	DUP	Uncertain	Uncertain		Uncertain	0
17	41,258,421	41,258,605	DEL	Uncertain	Likely Pathogenic	yes	Uncertain	0
17	41,258,421	41,258,605	DUP	Likely Pathogenic	Uncertain	yes	Uncertain	0
17	41,784,108	42,438,203	DUP	Uncertain	Uncertain		Uncertain	0
18	2,821,972	3,412,357	DUP	Uncertain	Uncertain		Uncertain	0
18	47,093,759	47,449,144	DUP	Uncertain	Uncertain		Uncertain	0
19	1,204,812	1,226,846	DUP	Uncertain	Uncertain		Uncertain	0
19	2,349,974	2,940,906	DUP	Uncertain	Uncertain		Uncertain	0
19	47,482,403	48,177,720	DEL	Uncertain	Likely Pathogenic	yes	Uncertain	0.15
20	14,618,195	14,744,154	DEL	Uncertain	Uncertain		Uncertain	0
22	20,109,547	21,290,949	DUP	Likely Pathogenic	Uncertain	yes	Uncertain	0
X	1,519,902	2,140,501	DUP	Uncertain	Uncertain		Uncertain	0
X	1,734,397	4,958,582	DUP	Uncertain	Uncertain		Uncertain	0
X	10,149,642	10,830,236	DUP	Uncertain	Uncertain		Uncertain	0
X	103,033,744	103,288,063	DUP	Pathogenic	Uncertain	yes	Uncertain	0

References

1. O’Leary, N. A. *et al.* Reference sequence (RefSeq) database at NCBI: current status, taxonomic expansion, and functional annotation. *Nucleic Acids Res.* **44**, D733–45 (2016).
2. Visel, A., Minovitsky, S., Dubchak, I. & Pennacchio, L. A. VISTA Enhancer Browser--a database of tissue-specific human enhancers. *Nucleic Acids Res.* **35**, D88–92 (2007).
3. Andersson, R. *et al.* An atlas of active enhancers across human cell types and tissues. *Nature* **507**, 455–461 (2014).
4. Zerbino, D. R., Wilder, S. P., Johnson, N., Juettemann, T. & Flicek, P. R. The ensembl regulatory build. *Genome Biol.* **16**, 56 (2015).
5. Kuhn, R. M., Haussler, D. & Kent, W. J. The UCSC genome browser and associated tools. *Brief. Bioinform.* **14**, 144–161 (2013).
6. Rehm, H. L. *et al.* ClinGen--the Clinical Genome Resource. *N. Engl. J. Med.* **372**, 2235–2242 (2015).
7. Firth, H. V. *et al.* DECIPHER: Database of Chromosomal Imbalance and Phenotype in Humans Using Ensembl Resources. *Am. J. Hum. Genet.* **84**, 524–533 (2009).
8. Lek, M. *et al.* Analysis of protein-coding genetic variation in 60,706 humans. *Nature* **536**, 285–291 (2016).
9. Collins, R. L. *et al.* A structural variation reference for medical and population genetics. *Nature* **581**, 444–451 (2020).
10. MacDonald, J. R., Ziman, R., Yuen, R. K. C., Feuk, L. & Scherer, S. W. The Database of Genomic Variants: a curated collection of structural variation in the human genome. *Nucleic Acids Res.* **42**, D986–92 (2014).
